# Leveraging interactive voice response technology to mitigate COVID-19 risk in refugee settlements in Uganda: Lessons learned implementing “Dial-COVID” a toll-free mobile phone symptom surveillance and information dissemination tool

**DOI:** 10.1371/journal.pone.0279373

**Published:** 2023-01-23

**Authors:** Robin E. Klabbers, Timothy R. Muwonge, Phuong Pham, Andrew Mujugira, Patrick Vinck, Sukanya Borthakur, Monisha Sharma, Numan Mohammed, Rosalind Parkes-Ratanshi, Connie Celum, Kelli N. O’Laughlin

**Affiliations:** 1 Department of Emergency Medicine, University of Washington, Seattle, Washington, United States of America; 2 Department of Global Health, University of Washington, Seattle, Washington, United States of America; 3 Infectious Diseases Institute, Makerere University, Kampala, Uganda; 4 Harvard Humanitarian Initiative, Harvard University, Cambridge, Massachusetts, United States of America; 5 Medical Teams International, Kampala, Uganda; 6 Viamo, Ghana; 7 Department of Psychiatry, University of Cambridge, Cambridge, United Kingdom; 8 Department of Epidemiology, University of Washington, Seattle, Washington, United States of America; 9 Department of Medicine, University of Washington, Seattle, Washington, United States of America; Vellore Institute of Technology: VIT University, INDIA

## Abstract

**Background:**

Persons living in refugee settlements in sub-Saharan Africa may be at increased risk for COVID-19 and experience barriers to accessing COVID-19 information. We aimed to evaluate the implementation of “Dial-COVID” a multi-lingual, toll free, telephone platform that uses interactive voice response (IVR) to track COVID-19 symptoms/exposure and disseminate COVID-19 health information in refugee settlements in Uganda. We hypothesized that IVR could provide an alternative way to screen for COVID-19 and communicate public health information to humanitarian populations when physical access and testing capacity were limited.

**Methods:**

The Dial-COVID IVR platform was created in ten languages and advertised by community health workers in refugee settlements for participants to call into toll free. In a recorded IVR symptom survey, participants were screened for COVID-19 symptoms/exposures and based on their responses, received tailored public health messages about COVID-19 risk mitigation in accordance with Uganda Ministry of Health guidelines. Here we report the challenges and lessons learned implementing this research during the pandemic.

**Results:**

Between February 2021 and March 2022, 15,465 calls were received by the Dial-COVID platform from all 31 refugee settlements in Uganda through which 6,913 symptom surveys were completed and 10,411 public health messages were disseminated in all study languages. Uptake of Dial-COVID fluctuated with the national COVID-19 caseload and was impacted by phone ownership and connectivity in refugee settlements. Intensified advertising efforts promoted Dial-COVID uptake. Flexibility to adapt IVR messages was contingent on translation capacity.

**Conclusion:**

Refugees living in refugee settlements across Uganda accessed Dial-COVID to share and obtain COVID-19 information suggesting that IVR holds potential for rapid information dissemination and screening of humanitarian populations during future infectious disease outbreaks and may be a valuable tool for routine public health programs. IVR adaptation flexibility and reach are influenced by language constraints and by contextual factors related to platform access.

**Registration details:**

World Pandemic Research Network– 490652.

## Introduction

The environment in refugee settlements may expose refugees and asylum seekers to increased risk of acquiring and transmitting infections, including severe acute respiratory syndrome coronavirus 2 (SARS-CoV-2), the virus causing coronavirus disease 2019 (COVID-19) [1]. Underdeveloped water and sanitation infrastructure, limited access to basic amenities (e.g., clean water, soap, hand sanitizer, cleaning supplies) and competing survival needs can undermine the ability to adhere to vital prevention and control measures (e.g., hand hygiene, quarantine, and self-isolation) [2–5], while population mobility and overcrowded living conditions may augment COVID-19 transmission [5–7]. These contextual factors in refugee camp settings culminate in a vulnerability to large-scale outbreaks where disease burden quickly exceeds healthcare capacity [8]. Reaching populations in refugee settlements with public health messaging to mitigate the potentially heightened COVID-19 risk is challenged by language diversity, varying literacy levels, and lack of internet connectivity, resulting in limited access to information for individuals living in these contexts [1, 5, 9–12].

Globally, mobile phone and internet-based tools developed or adapted for COVID-19 have been successfully deployed to identify individuals with probable COVID-19 and measure community disease burden [13–17]. Symptom trackers have been used to map COVID-19 spread, inform outbreak projection models, and target resources and public health messaging [15, 16, 18]. These tools have been praised for their agility and scalability which allowed for a rapid response to the pandemic [19]. However, while these tools have been effective in locations where the internet is readily accessible, they are likely less effective in humanitarian settings where there is low smartphone ownership and limited internet connectivity or wireless infrastructure. Telephone-based interventions that do not require the internet may be an effective alternative. Investigators are currently researching an SMS-based intervention in a cluster randomized controlled trial among refugee youth in Kampala in which SMS messages are used to check-in with study participants and share COVID-19 information with the aim of increasing COVID-19 prevention practices [20]. Considering the varying levels of literacy among refugee populations, an intervention that relies on SMS messages with text may not be optimal to share and collect information [9, 12].

Telephone-based interactive voice response (IVR), in which participants respond to recorded audio questions by entering numbers on a simple (analogue) phone keypad, may be a promising solution to the aforementioned challenges [21]. When offered free of charge in the appropriate language, telephone-based IVR can overcome many barriers refugees face in accessing COVID-19 information. IVR makes use of audio messages and therefore does not rely on a high level of literacy. Individuals do not need to own a smartphone or have access to internet connectivity to use IVR and can interact with IVR surveys through phone calls. Compared to traditional methods of data collection such as door-to-door household surveys, this tool requires minimal in-person interaction or set-up time making it especially useful in emergency and conflict settings where physical contact can be challenging [22]. IVR has previously been used in remote and humanitarian contexts including in Afghanistan, the Democratic Republic of the Congo (DRC), Ghana, Haiti, Niger, and Somalia to collect and disseminate information on a host of topics including food insecurity, cash transfers, aid presence, health behavior, and violence prevention [22–29]. It is a flexible system that lends itself to a range of different content and has effectively been used in low- and middle-income countries for both information collection and dissemination during the COVID-19 pandemic [30–34]. IVR can be used as a standalone tool, or can be combined with other tools/methods to augment data collection and dissemination as was done by the International Organization for Migration (IOM) in Cox’s Bazaar, Bangladesh, where participants were screened using an IVR survey to identify those eligible to receive a call back with a specific needs assessment survey [23, 24, 35]. IVR has been shown to be acceptable and useful in qualitative studies and a mobile phone-based IVR health information and surveillance system in Ghana was demonstrated to support user healthcare decision-making and disease awareness [36]. IVR is being explored as a data collection tool to reach groups that are often missed using conventional techniques with promising initial results [37].

IVR has been used in many applications for both regular health programming and in emergency contexts. Its remote nature, minimal infrastructure requirements, and adaptability make it an attractive tool for disseminating and collecting information in humanitarian contexts. It is unknown whether the characteristics of this tool can be leveraged during an emerging pandemic to surveil for COVID-19 symptoms and reach refugee populations with COVID-19 risk mitigation information. We aimed to evaluate the feasibility of using “Dial-COVID”, a telephone platform that uses IVR to screen for COVID-19 symptoms and exposures and to disseminate COVID-19 information to refugee populations with limited literacy in Uganda. In this paper, we describe the Dial-COVID study methodology and the lessons learned implementing this research to enhance the future use of IVR applications for humanitarian populations.

## Materials and methods

### Study aims and objectives

Dial-COVID was a proof-of-concept study to determine whether phone-based IVR technology could feasibly be used to collect COVID-19 symptom and exposure data and disseminate COVID-19 information and risk mitigation messages in refugee settlements in Uganda in the context of a global pandemic. Based on the Health Belief Model, we hoped that improved access to COVID-19 information would ultimately change individual perceptions of COVID-19 susceptibility and severity and function as a cue to action to adopt appropriate protection behaviors [38]. We aimed to assess the uptake of the Dial-COVID symptom survey and Dial-COVID’s ability to disseminate information in refugee settlements. IVR was selected as the data collection methodology since it eliminates the need for face-to-face interactions while maintaining large geographic reach. This was crucial as in-person data collection in refugee settlements throughout Uganda during the COVID-19 pandemic was challenged by COVID-19 transmission risk and intermittent travel restrictions.

### Formation of the research consortium

The Dial-COVID study was designed and implemented by a research consortium consisting of partners in academia (University of Washington, Makerere University Infectious Diseases Institute, Harvard Humanitarian Initiative), a non-governmental organization (Medical Teams International), and a telecommunication company (Viamo), all with extensive experience in Uganda. Collaboration with and approval from relevant stakeholders was sought including the Uganda Ministry of Health and the United Nations High Commissioner for Refugees (UNHCR).

### Study setting

**Fig 1:**
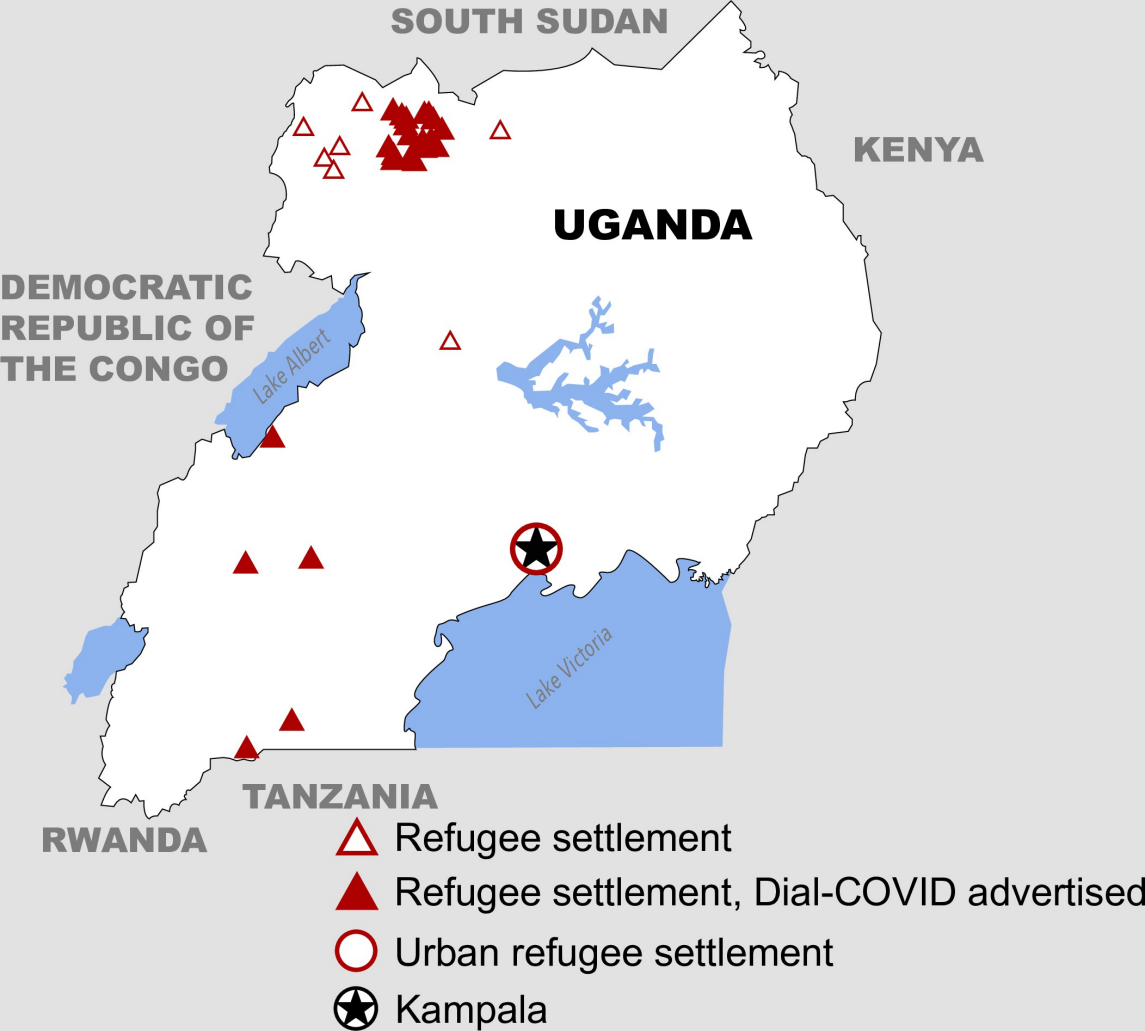
Map of refugee settlements in Uganda including Dial-COVID advertisement sites. Refugee settlements where Dial-COVID was advertised include Nakivale, Oruchinga, Rwamwanja, Kyaka II, Kyangwali, Oliji*, Maaji I*, Maaji II*, Maaji III*, Mungula*, Mungula II*, Ayilo I*, Ayilo II*, Alere 2*, Boroli*, Olua I*, Olua II*, Pagrinya*, Nyumanzi*, Elema*, Baratuku*, Agojo*, Mirieyi*, and Palorinya. *Referred to collectively as Adjumani Refugee Settlement.

Dial-COVID was conducted in Uganda, the third largest refugee hosting nation in the world, with 1.5 million refugees [39]. The vast majority of refugees in Uganda (94%) live in refugee settlements [40]. Northern Uganda has the largest refugee population (57%), followed by southwestern (24%), and central Uganda (13%). The remaining 6% of refugees live outside of refugee settlements in urban centers, most commonly in the capital city, Kampala. South Sudanese refugees make up the largest refugee subpopulation (943,991; 61%), followed by refugees from the Democratic Republic of the Congo (DRC) (449,863; 29%), Burundi (51,899; 3%), and Somalia (50,290; 3%). Other subpopulations include refugees from Rwanda, Eritrea, Ethiopia, and Sudan [41]. Dial-COVID was advertised in the refugee settlements in Uganda where Medical Teams International is the health implementing partner; this included 24 (77%) of the 31 refugee settlements in Uganda, home to 888,978 (55.7%) refugees (Fig 1). Refugee settlements in Uganda are not exclusively home to refugees and asylum seekers; an unknown number of Ugandan nationals live within or close to the settlement boundaries benefiting from the infrastructure and resources provided by humanitarian programs.

### Dial-COVID interactive voice response (IVR) platform

Two Ugandan phone numbers were secured in partnership with Viamo (https://viamo.io), a telecommunications company founded in Ghana with representation in more than 20 markets in Africa and Asia. One number was advertised in refugee settlements in Northern Ugandan and one in Southern Uganda. Both numbers were linked to an online platform with the ability to send out IVR surveys. The platform was designed so that when a person “beeped” the line (i.e., called in to the platform by dialing the Dial-COVID number and immediately hung up to avoid call charges), a free return call was generated; this is a common method used in Uganda to ensure a toll-free call. The return call prompted the Dial-COVID symptom survey; a series of prerecorded IVR messages and questions about COVID-19 symptoms and exposures that participants responded to using their phone keypad. In addition, the platform was programmed so that a batch of outbound calls containing the same Dial-COVID symptom survey was pushed out monthly from the Dial-COVID platform to Ugandan phone numbers generated through random digit dialing (RDD) to provide a nationwide comparison sample.

### Dial-COVID symptom survey and information dissemination instrument design

The Dial-COVID symptom survey questions were scripted based on the “symptoms, clinical course, past medical history and social history” section of the Centers for Disease Control and Prevention Human Infection with 2019 Novel Coronavirus Person Under Investigation (PUI) and Case Report Form [42] (S1 Appendix). The two public health messages included in the symptom survey were scripted in accordance with Uganda Ministry of Health guidelines [43, 44]. Scripts were reviewed by the research team and collaborators in the study setting for appropriateness and iteratively were refined. Scripts were then translated by certified translators into Arabic, Dinka, Kakwa, Kinyarwanda, Kiswahili, Lugbara, Runyankore, and Somali—the nine languages most commonly spoken by refugees in Uganda, and into Luganda, which is commonly spoken by Ugandan nationals. Accuracy and appropriateness of translations were assessed by native speakers from the local community fluent in English and one of the study languages before audio recording of the scripts by Viamo. Quality checks were performed for all recordings. Recordings for the survey languages were split geographically and allocated to the North phone number (Arabic, Dinka, English, Kakwa, Kiswahili, Lugbara, Runyankore), the South phone number (English, Kinyarwanda, Kiswahili, Runyankore, Somali), and the RDD line (English, Luganda, Kiswahili, Runyankore, Lugbara) based on the predominant languages in the respective geographic areas so participants would not be deterred by a long list of language options when first interacting with the platform.

### Dial-COVID symptom survey content

Participants interacting with the Dial-COVID symptom survey were first asked to select their language of choice. A general message followed explaining IVR and providing information on the Dial-COVID study after which callers were asked if they would like to continue. Consent was implied through voluntary participation after eligibility was assessed through recorded questions at the beginning of the survey. The option to hear COVID-19 related information was given regardless of survey participation. Individuals eligible and willing to participate received the Dial-COVID symptom survey which included questions about sociodemographic information (8 questions), pre-existing health conditions (8 questions), COVID-19 symptoms (11 questions), COVID-19 exposures (1 question), quarantine/hospitalization status (1 question), and health workers status (1 question) (S1 Appendix). Additionally, participants were asked whether they consented to being contacted for additional research. Depending on the answers provided in the symptom survey, participants received one of two public health messages at the end of the survey. Participants reporting COVID-19 symptoms or exposure received a public health message advising them to self-isolate and seek medical attention if they developed severe symptoms including trouble breathing or chest pain. The message also included a toll-free Ministry of Health phone number which participants could call to discuss symptoms/exposures and possible next steps. Participants reporting no COVID-19 symptoms or exposures, or those who did not wish to participate in the symptom survey, received general risk mitigation guidelines including staying home when possible, physical distancing, wearing a facemask, and appropriate hand hygiene. All participants received COVID-19 transmission information and a message aimed to minimize COVID-19 stigma.

A subset of participants consenting to being contacted for future COVID-19 research at the end of the Dial-COVID symptom survey were enrolled in additional research including COVID-19 polymerase chain reaction testing to determine if a COVID-19 prediction algorithm could be designed; a three-round longitudinal IVR survey assessing COVID-19 knowledge, risk perception, perspectives on mitigation strategy adoption, and vaccination willingness/hesitancy; and a one-time in-depth qualitative interview exploring barriers and facilitators of COVID-19 prevention measure adoption and vaccination willingness/hesitancy (related manuscripts forthcoming).

### Participant recruitment and eligibility criteria

**Fig 2:**
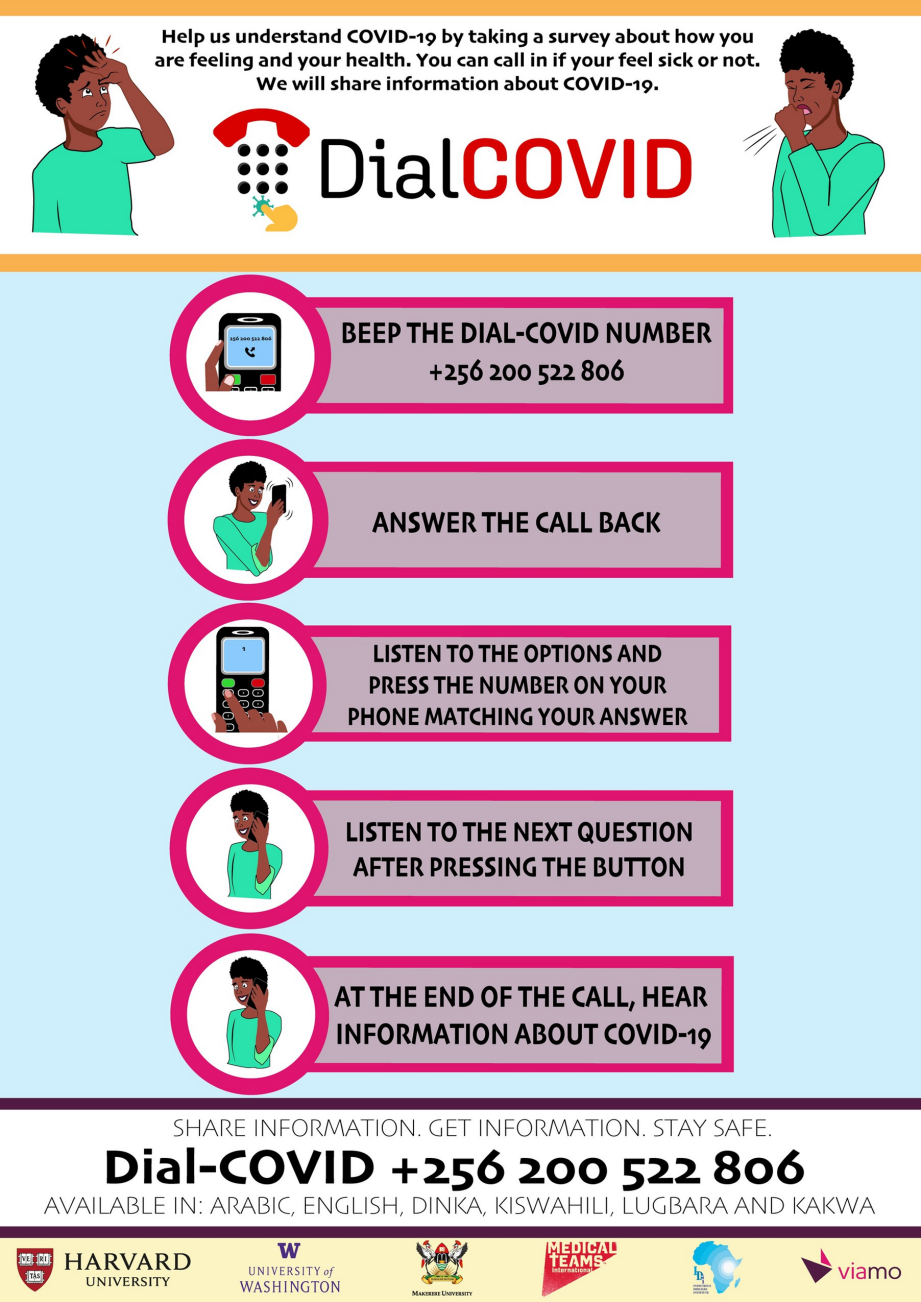
Dial-COVID poster advertisement example.

To promote platform uptake, the Dial-COVID phone numbers were advertised in refugee settlements. Using a train-the-trainer model [45], Medical Teams International Public Health Officers and Health Assistants were trained in Dial-COVID study operations. These Health Officers and Health Assistants then trained Village Health Teams (community health workers selected and trained by Medical Teams International to promote health at the community level in the refugee settlements) to drive advertising efforts and support study uptake. Advertising efforts include posting of instructive posters and flyers in all study languages (Fig 2), megaphone announcements at places of congregation such as at food distribution, radio spots, and door-to-door publicizing of the platform. When necessary, Village Health Teams assisted participants in calling into the platform. As the Dial-COVID phone numbers were advertised exclusively in refugee settlements, it was expected that the majority of participants calling into Dial-COVID would be refugees, and some might be Ugandan nationals living in and around refugee settlements. Individuals were considered eligible for participation if they called the Dial-COVID number, were willing to complete the Dial-COVID symptom survey, were ≥18 years of age, and spoke one of the study languages. We aimed to obtain a sample of 10,000 symptom surveys completed by call-in participants.

As a comparison group, a nationally representative sample was recruited through RDD of Ugandan phone numbers by Viamo’s systems. RDD calls with the Dial-COVID symptom survey were programmed to be pushed out monthly for six months with the aim of obtaining 1,500 completed surveys per month (9,000 total). As phone numbers were generated randomly, the comparison sample was expected to reflect the general population in Uganda and include Ugandan nationals and refugees living throughout Uganda within and outside of refugee settlements. Eligibility criteria included age ≥18 years, willingness to complete the symptom survey, and command of one of the study languages. To minimize privacy concerns, when individuals answered the Dial-COVID call, they were informed that their number was randomly generated. Participants were not charged for answering the call.

### Support of Dial-COVID implementation

To support study implementation, weekly meetings were held that were attended by representatives from Viamo, Medical Teams International and researchers from the University of Washington, Harvard University, and Makerere University Infectious Diseases Institute. During these meetings, call volume and technical functionality of the platform were discussed, updates and feedback were given from the field, and any arising issues were addressed by making adaptations to study implementation as needed. In addition, monthly research consortium meetings were held for all Dial-COVID stakeholders to promote engagement and foster communication. Meetings consisted of updates and a presentation of high-level findings followed by either a guided discussion or an open forum for stakeholders to provide input.

### Ethical considerations

Ethical approval to conduct this study was obtained from the University of Washington Human Subjects Division (STUDY00010663, 8/17/2020), the Makerere University School of Health Sciences Research Ethics Committee (SHSREC REF No. 2020–14, 7/20/2020), and the Mass General Brigham Institutional Review Board (2020P002593, 9/9/2020). Consistent with the national guidelines in Uganda, clearance to conduct this study was also obtained from Uganda National Council for Science and Technology (HS901ES, 11/4/2020).

### Inclusivity in global research

Additional information regarding the ethical, cultural, and scientific considerations specific to inclusivity in global health is included in the Supporting Information (S2 Appendix).

## Results

The Dial-COVID platform launched in February 2021, between the first and the second COVID-19 wave in Uganda and was active through April 2022. During this time, 15,465 calls were received and 9,912 RDD calls were sent out by the Dial-COVID platform. A total of 6,913 Dial-COVID symptom surveys were completed by call-in participants and 1,413 by RDD participants. Though Dial-COVID, public health information about COVID-19 was shared during 10,411 call-in encounters and 3,737 RDD encounters. Calls were conducted in all available study languages and were received by the platform from participants in all four regions of Uganda and all 31 refugee settlements, including those refugee settlements where Dial-COVID was not advertised (Table 1). Detailed findings regarding population composition and symptom survey results for the two groups will be shared in a separate manuscript.

**Table 1:** Language and geographic distribution of Dial-COVID call-in encounters.

	Call-in encounters (Total = 15,465) N (%)^a^
**Language (N = 15,464)**	
Arabic	208 (1.3%)
Dinka	239 (1.5%)
English	1,273 (8.2%)
Kakwa	80 (0.5%)
Kinyarwanda	5,358 (34.6%)
Kiswahili	7,467 (48.3%)
Lugbara	47 (0.3%)
Runyankore	692 (4.5%)
Somali	100 (0.6%)
**Region (N = 10,576)**	
Northern or West Nile region	846 (8.0%)
Central or East Central region	1,463 (13.8%)
Eastern region	838 (7.9%)
Western or South western region	7,429 (70.2%)
**Refugee Settlement (N = 8,549)**	
Adjumani Refugee Settlement^b^	210 (2.5%)
Bidi Bidi Refugee Settlement	27 (0.3%)
Imvepi Refugee Settlement	15 (0.2%)
Kampala Refugee Settlement	781 (9.1%)
Kiryandongo Refugee Settlement	254 (3.0%)
Kyagwali Refugee Settlement	470 (5.5%)
Kyaka II Refugee Settlement	2,342 (27.4%)
Lobule Refugee Settlement	51 (0.6%)
Nakivale Refugee Settlement	344 (4.0%)
Oruchinga Refugee Settlement	436 (5.1%)
Palabek Refugee Settlement	37 (0.4%)
Palorinya Refugee Settlement	163 (1.9%)
Rhino Camp Refugee Settlement	41 (0.5%)
Rwamwanja Refugee Settlement	3,378 (39.5%)

^a^ Of those participants answering the relevant question. Excludes participants who did not receive the relevant question or opted to listen to COVID-19 public health information only.

^b^ Collective term used to refer to the Refugee Settlements Oliji, Maaji I, Maaji II, Maaji III, Mungula, Mungula II, Ayilo I, Ayilo II, Alere 2, Boroli, Olua I, Olua II, Pagrinya, Nyumanzi, Elema, Baratuku, Agojo, and Mirieyi.

Throughout the study, iterative adaptations were made to address implementation challenges and optimize IVR functionality (Table 2). Platform uptake varied by refugee settlement and call volume fluctuated over time. In close collaboration with Medical Teams International Village Health Teams working in the refugee settlements and colleagues from Viamo operating the Dial-COVID platform, these issues were analyzed. Variation in uptake was likely a culmination of differences in Dial-COVID awareness and barriers to platform access–including the degree of phone and Ugandan SIM card ownership, possession of airtime (credit needed to place a call), and network connectivity.

**Table 2:** Lessons learned implementing Dial-COVID.

Challenge	Lesson learned	Proposed solution
Troubleshooting and understanding unexpected data	Having personnel on the ground to provide feedback was imperative to ensure tool effectiveness	Have a small number of support staff in the field or collaborate closely with professionals in the target setting
Ensuring platform uptake by study participants	Uptake fluctuated with pandemic coverage in the media and COVID-19 case numbers	Provide continuous advertisement through multiple mediums
		Use outbound calls to limit participant effort required
Accessing the platform requires a phone, SIM card and positive airtime balance	Phone and SIM card access were not ubiquitous particularly in refugee settlements	Make phones and airtime available at a central location for those without personal access
	Airtime debt was common	Facilitate registration and Ugandan SIM card acquisition for newly arrived refugees
Connecting to the network	Both the local network connection and the platform server were not reliable	Collaborate with the telecommunications company supporting the project to identify and solve network issues in a timely manner
		Promote designated areas in the refugee settlement with good connectivity to place calls to the platform
Adapting the Dial-COVID data collection tools to shifting priorities in the COVID-19 pandemic	Offering a multilingual platform came at the expense of adaptation agility	Have a dependable roster of available qualified translators in place before study initiation and maintain this roster throughout the study

During weekly meetings, solutions to challenges were discussed and subsequently implemented. To improve awareness, the Dial-COVID advertising campaign was bolstered with the help of Medical Teams International Village Health Teams and expanded to different mediums. In addition, a one-time airtime distribution was provided to those individuals without airtime to facilitate calling in to Dial-COVID. Close communication between staff in the field, the research team and the telecommunications provider Viamo during weekly implementation meetings ensured that any network issues with the platform were quickly identified and addressed when possible.

As priorities in the COVID-19 pandemic shifted, it was important to incorporate information about COVID-19 vaccination into the Dial-COVID messaging. Finding certified translators to translate these messages into the nine study languages (excluding English) and check recordings proved challenging as a result of pandemic-related staffing shortages among translation services. Alternative sources of translation expertise were considered including embassy staff and the network of the research team to minimize study delays.

To facilitate future IVR research in humanitarian contexts, we summarize implementation challenges, early lessons learned initiating Dial-COVID, and proposed study adaptations in Table 2.

## Discussion

Refugees living in all refugee settlements and all districts of Uganda actively engaged with Dial-COVID, a phone-based, multi-lingual, toll free IVR platform that was advertised in refugee settlements, accessing the tool in all available study languages. With the support of community health workers for platform advertisement and troubleshooting, IVR enabled remote screening of persons in refugee settlements for COVID-19 symptoms and exposure and dissemination of COVID-19 risk mitigation messages at a time when COVID-19 testing capacity was limited and restrictions on movement challenged field visits. Uptake of Dial-COVID extended beyond the refugee settlements where the platform was advertised, suggesting information about the Dial-COVID platform was spread by word of mouth and a clear demand existed among the refugee population for COVID-19 risk mitigation information. The implementation of Dial-COVID was not without challenges and valuable lessons were learned that can aid others considering the use of IVR tools in humanitarian contexts.

### Personnel in the field facilitate data collection and information dissemination

Having personnel on the ground was invaluable for troubleshooting and understanding data in the early phases of the research. Close communication with the Village Health Teams driving advertising efforts in refugee settlements allowed for real-time feedback regarding difficulties in accessing the platform or confusion regarding advertising material. Findings from other IVR studies have noted that explaining the IVR process before exposure to the technology improved response rates [46].’

### Platform uptake fluctuates with media coverage and public interest

Uganda reported its first COVID-19 case on March 21^st^, 2020, and experienced the first, second, and third waves of the COVID-19 pandemic between August 2020 –January 2021, May 2021 –August 2021, and December 2021 –February 2022, respectively [47, 48]. Uptake of the Dial-COVID symptom survey fluctuated with the degree to which COVID-19 was at the forefront of public interest. Other information campaigns competed for public interest (e.g., the national elections, other infectious disease outbreaks) and seemed to hinder uptake. To address this, we aimed to have continuous advertising through multiple mediums (e.g., radio spots, megaphone messages, posters). However, national restrictions on congregation, movement, and travel, likely attenuated the effectiveness of these efforts. Further, women and the elderly, who spend more time in and around the home, were likely not reached by these messaging strategies. For future IVR interventions contingent on participant initiative to be successful, it is imperative that a robust, adequately funded, continuous advertising strategy is in place. Performance auditing and feedback provision as well as repeated training and distribution of advertising materials to teams in charge of uptake promotion could be used to facilitate this. An alternative for future interventions could be to use direct text messaging to all numbers in the geographic region to promote platform awareness rather than relying on in-person advertisement.

### Access to call-in prerequisites is not ubiquitous in refugee settlements

Feedback from Village Health Teams revealed that not everyone living in the settlements where Dial-COVID was advertised was able to place a call to the platform. Lack of access to a mobile phone or SIM card or insufficient ‘airtime’ credit formed barriers to platform uptake for some individuals. Data on phone ownership among the refugee population in Uganda is limited. One study conducted in Bidi Bidi, the largest refugee settlement in Uganda (not covered by Dial-COVID advertising) in 2018 by the United Nations Capital Development Fund and DanChurchAid, estimated that 73% of men and 44% of women had a phone, highlighting that this barrier likely is not distributed equally [49]. While interacting with the Dial-COVID platform was toll-free, there were certain prerequisites set by Ugandan mobile network operators in order to benefit from this service. First, to receive a toll-free call back, the call had to be placed using a Ugandan SIM card which not all refugees and asylum seekers who had recently entered the country possessed. Facilitating registration and Ugandan SIM card acquisition for newly arrived refugees could improve reach in the future. Second, some mobile network operators required callers to have a positive airtime credit balance to place a call. An attempt was made to circumvent this issue during the study by having Village Health Teams distribute a small airtime credit of UGX 200 (~$0.06, €0.05) to participants without airtime, but this solution was met with additional challenges. It is common in Uganda to buy airtime on credit, and any new funds that are added to an account with an airtime debt are automatically put towards paying the debt. Frequently, the airtime distributed to participants by Village Health Teams was immediately deducted and was not sufficient to overcome the debt balance, leaving participants still unable to place a call. Similar to Dial-COVID, challenges surrounding mobile network operators have also been reported in other settings where IVR has been implemented and are often difficult to address [23].

### Network connectivity may be unreliable in humanitarian settings

In Northern Uganda, calls were frequently disconnected or dropped and at times individuals were unable to place calls. Problems with network connectivity is a common challenge in these remote contexts and have been reported in other IVR initiatives as an explanation for incomplete surveys [50]. One solution to improve connectivity, which some people in Uganda choose, is to carry multiple SIM cards and swap between them depending on their location as network coverage varies geographically per mobile network operator. While this improves connectivity, SIM card switching posed a challenge for study recruitment and participant follow up. SIM cards were sometimes not in use at the time of recruitment calls. This could however also have been the result of phones being off to preserve battery, which is common in this setting with regular power outages and lack of access to charging. SIM card swapping and related practices like phone sharing are not limited to the Ugandan context and have limited the effectiveness of an IVR intervention deployed in Haiti [51].

Dial-COVID uptake was also contingent on the platform being operational and accessible, and was therefore vulnerable to issues with the hosting network. In the first few months of data collection, the phone network was not operational on multiple occasions due to server issues preventing study recruitment during these times. Close collaboration with Viamo, the telecommunications company supporting Dial-COVID, was essential to quickly address these issues.

### IVR message responsiveness depends on translation capacity

While not the objective of this research at the outset, a better understanding of COVID-19 vaccines was considered highly relevant to the COVID-19 response when vaccines became available in the spring of 2021. We adapted our study instruments, including a longitudinal IVR survey and qualitative interview guide, to include questions about COVID-19 vaccination knowledge and willingness/hesitancy. While IVR and mobile phone-based data collection tools are favored for their agility, in the case of this study, adaptations of study instruments required translations, recordings, and review in nine different languages, making this an arduous and lengthy process. Further, due to staffing issues among translation services during the COVID-19 pandemic, language translations were delayed. Having a dependable roster of qualified translators in place before study initiation and throughout the study may allow for swift instrument adaptation during a dynamic pandemic. If IVR is to be deployed more widely in the future for public health response, this translator roster could take on a more permanent structure and could be folded into international organizations so that these human resources can be shared among public health initiatives. In other humanitarian contexts where IVR has been deployed, careful scripting of IVR messages was also considered more time-consuming than initially anticipated [52].

### IVR characteristics should be considered when designing data collection and information dissemination tools

Inherent characteristics of IVR make it more suitable for certain types of data collection than others. First, there are a limited number of question and answer options that an IVR survey can contain. Response fatigue may occur more quickly when listening to audio recordings than with traditional survey modalities where an administrator and visual aids are present to promote participant engagement and sustained attention. In Dial-COVID, information fatigue motivated the choice to create two Dial-COVID numbers and split the languages by region instead of requiring participants to listen to an exhaustive list of possible language options when first interacting with the platform. Second, when implied consent (as opposed to verbal or written consent) is used, as was done in Dial-COVID, participant confidentiality and the type of identifiers that can be collected should be considered. In Dial-COVID, all survey answer options were categorical, as opposed to free input, impacting the granularity of data we were able to collect and our ability to link IVR data to a specific individual in a context where phone sharing is common.

### Limitations

This research was designed to examine the experience of refugees living in refugee settlements, as opposed to refugees living in urban centers (although RDD may reach a number of refugees living in urban centers). The unique challenges of the living arrangements in refugee settlements for COVID-19 risk mitigation motivated this focus. As such, the results obtained through the Dial-COVID platform may not reflect the experience of all refugees in Uganda. Additionally, the nature of IVR data collection adds bias by limiting enrollment to those with phone access. Phone ownership is associated with higher literacy and socioeconomic status and results obtained in the Dial-COVID study may therefore not be generalizable to the general refugee population [49]. Despite this limitation, over 15,000 individual calls were received by the Dial-COVID platform, and while a certain degree of technological savviness and phone access are required to call into the Dial-COVID platform, the benefits of any information individuals obtain through interacting with Dial-COVID can extend to an individual’s household and community through information sharing. Analysis of the demographic characteristics of the participants will help determine whether specific groups were not reached. Future research should focus on assessing IVR platform interaction from the participant’s perspective with the goal of optimizing reach and ease of use.

## Conclusions

Phone-based interactive voice response (IVR) offers researchers and community engagement programs a viable tool to collect data from and disseminate critical public health information to multi-lingual humanitarian populations with limited literacy when physical access or resources are limited. IVR requires little infrastructure, can be rapidly deployed in many languages and is easily applied to new public health challenges. Challenges encountered during Dial-COVID study implementation highlight that the inclusivity and effectiveness of IVR in humanitarian settings will depend on the ability to address context-specific barriers to accessing IVR tools. The lessons learned in Uganda can help optimize the design of IVR applications for future public health emergencies.

## Supporting information

S1 Appendix Dial-COVID symptom survey. (DOCX)

S2 Appendix Checklist. (DOCX)
