# Supplementary material for: Leveraging interactive voice response technology to mitigate COVID-19 risk in refugee settlements in Uganda: Lessons learned implementing “Dial-COVID” a toll-free mobile phone symptom surveillance and information dissemination tool
Source: PLoS One. 2023 Jan 23;18(1):e0279373. doi: 10.1371/journal.pone.0279373 (PMC9870132; doi:10.1371/journal.pone.0279373)
Supplement: S1 Appendix — (DOCX) [file pone.0279373.s001.docx]

**Appendix A**

**Dial-COVID Symptom Survey**

| North | South | | RDD |
| --- | --- | --- | --- |
| For Arabic, press 1  For English, press 2  For Dinka, press 3  For Kiswahili, press 4  For Lugbara, press 5  For Kakwa, press 6 | For Kiswahili, press 1  For Kinyarwanda, press 2  For English, press 3  For Runyankore, press 4  For Somali, press 5 | | For English, press 1  For Luganda, press 2  For Kiswahili, press 3  For Runyankore, press 4  For Lugbara, press 5 |
| Hello, and thank you for calling Dial-COVID.  This call is from Makerere and the University of Washington in the USA.  We hope you will take part in a 10-minute survey that will help us understand COVID-19 better.  Your responses to the survey will be used only for research purposes.  Your name and personal information will remain confidential.  You do not have to do the survey if you do not want to.  After you finish, we will share useful information with you about COVID-19.  To answer the survey questions, select the number that corresponds to your answer on your telephone keypad.  Please listen carefully to the whole question before answering.  You can listen to the question again if you did not hear it well the first time.  There are no right or wrong answers; please answer honestly.  Thank you for your help. For the following questions, if you want to hear the question again, press 0. | | | Hello, your number was selected at random to receive this call.  This call is from Makerere and the University of Washington in the USA.  We hope you will take part in a 10-minute survey that will help us understand COVID-19 better.  Your responses to the survey will be used only for research purposes.  Your name and personal information will remain confidential.  You do not have to do the survey if you do not want to.  After you finish, we will share useful information with you about COVID-19.  To answer the survey questions, select the number that corresponds to your answer on your telephone keypad.  Please listen carefully to the whole question before answering.  You can listen to the question again if you did not hear it well the first time.  There are no right or wrong answers; please answer honestly.  Thank you for your help.  For the following questions, if you want to hear the question again, press 0. |
| Press 1 if you agree to participate in the survey. [Continue to question 1]  Press 2 if you do not want to participate in the survey but still want to hear information about COVID-19. [Go to PUBLIC HEALTH MESSAGE]  Hang up if you do not want to participate in the survey and do not want to hear information about COVID-19. | | | |
| 1. If you are younger than 18 years, press 1 [Continue to ineligible message 1] If you are between 18 to 25 years of age, press 2.  If you are between 26 to 35, press 3.  If you are between 36 to 55, press 4.  If you are 56 years old or older, press 5.  If you prefer not to answer, press 6. | | | |
|  | | [INELIGIBLE MESSAGE 1]  We are sorry, you are not old enough to participate in this research. If you would still want to hear information about COVID-19, Press 1. [Go to PUBLIC HEALTH MESSAGE] | |
| 1. If you are a female, press 1.  If you are a male, press 2.  If you are other or prefer not to answer, press 3. | | | |
| 1. If you have not attended school, press 1.   If you attended primary school but did not finish Primary 7, press 2.  If you completed Primary 7, press 3.  If you attend some school after Primary 7, press 4.  If you prefer not to answer, press 5. | | | |
| 1. If you are a Ugandan national and are not displaced, Press 1.   If you are a Ugandan national and are internally displaced, Press 2.  If you are a refugee, Press 3.  If you are an asylum seeker, Press 4.  If you are from a country other than Uganda but not a refugee, Press 5.  If you prefer to not answer, Press 6. | | | |
| 1. Do you live in a refugee settlement?   Press 1 for Yes  Press 2 for No | | | |
| 1. In which region are you currently residing?   If you reside in the Western or Southwestern region, press 1  If you reside in the Central or East Central region, press 2  If you reside in the Northern or West Nile region, press 3  If you reside in the Eastern region, press 4 | | | |
| Western/South western | Central/East Central | | Northern/West Nile |
| 7A. In which District do you reside?  If you reside in Kikuube District, press 1  If you reside in Isingiro District, press 2  If you reside in Kyegegwa District, press 3  If you reside in Kamwenge District, press 4  If you reside in another District, press 5 | 7B. In which District do you reside?  If you live in Kiryandongo District, press 1  If you live in Kampala District, press 2  If you reside in another District, press 3 | | 7C. In which District do you reside?  If you live in Adjumani District, press 1  If you live in Obongi District, press 2  If you live in Koboko District, press 3  If you live in Yumbe District, press 4  If you live in Arua District, press 5  If you live in Lamwo District, press 6  If you reside in another District, press 7 |
| [If selected Yes for question 5 refugee settlement]  8A. Specify the refugee settlement where you reside.  If you live Kyagwali Refugee Settlement, press 1  If you live in Nakivale Refugee Settlement, press 2  If you live in Oruchinga Refugee Settlement, press 3  If you live in Kyaka II Refugee Settlement, press 4  If you live in Rwamwanja Refugee Settlement, press 5 | [If selected Yes for question 5 refugee settlement]  8B. Specify the refugee settlement where you reside.  If you live in Kiryandongo Refugee Settlement, press 1  If you live in Kampala Refugee Settlement, press 2 | | [If selected Yes for question 5 refugee settlement]  8C. Specify the refugee settlement where you reside.  If you live in Adjumani Refugee Settlement, press 1  If you live in Palorinya Refugee Settlement, press 2  If you live in Lobule Refugee Settlement, press 3  If you live in Bidi Bidi Refugee Settlement, press 4  If you live in Rhino Camp Refugee Settlement, press 5  If you live in Imvepi Refugee Settlement, press 6  If you live in Palabek Refugee Settlement, press 7 |
| S1. In the last 2 weeks Have you felt feverish or had chills?  Press 1 for Yes  Press 2 for No | | | |
| S2. In the last 2 weeks have you had a new or worsening cough?  Press 1 for Yes  Press 2 for No | | | |
|  | [If S2 is Yes]  S3. In the last 2 weeks Do you have a new or worsening persistent cough  (coughing a lot for more than an hour, or 3 or more episodes in 24 hours)?  Press 1 for Yes  Press 2 for No | | |
| S4. [In the last 2 weeks] Have you experienced shortness of breath or pain or tightness in your chest?  Press 1 for Yes  Press 2 for No | | | |
| S5. [In the last 2 weeks] have you experienced the loss of your ability to smell or taste?  Press 1 for Yes  Press 2 for No | | | |
| S6. [In the last 2 weeks] Have you experienced unusually strong muscle pains?  Press 1 for Yes  Press 2 for No | | | |
| S7. [In the last 2 weeks] Have you experienced a sore throat or an unusually hoarse voice?  Press 1 for Yes  Press 2 for No | | | |
| S8. Are you experiencing unusual fatigue?  Press 1 for Yes  Press 2 for No | | | |
|  | [If S8 is Yes]  S9. Have you been feeling so tired that you struggle to get out of bed?  Press 1 for Yes  Press 2 for No | | |
| S10. Do you have diarrhea or stool that is runny or watery?  Press 1 for Yes  Press 2 for No | | | |
| S11. Is your body telling you it does not feel hungry?  Press 1 for Yes  Press 2 for No | | | |
| R1. Do you have chronic health problems that require medications, treatments or medical support?  Press 1 for Yes  Press 2 for No | | | |
| R2. Do you have a prior history of respiratory problems such as asthma or lung disease?  Press 1 for Yes  Press 2 for No | | | |
| R3. Do you currently smoke tobacco on a daily basis, less than daily, or not at all?  Press 1 if you smoke Daily  Press 2 if you smoke Less than daily  Press 3 if you do Not smoke at all | | | |
| R4a. Do you have diabetes?  Press 1 for Yes  Press 2 for No | | | |
| R4b. Do you have hypertension?  Press 1 for Yes  Press 2 for No | | | |
| R4c. Do you have heart disease?  Press 1 for Yes  Press 2 for No | | | |
| R5a. Do you have a medical condition that suppresses your immune system such as HIV?  Press 1 for Yes  Press 2 for No | | | |
| R5b. Do you take a medication that suppresses your immune system such as treatment for cancer?  Press 1 for Yes  Press 2 for No | | | |
| R6. In the last month, have you been in close contact with someone diagnosed with COVID-19  or a person who is sick with new respiratory symptoms?  Press 1 for Yes  Press 2 for No  Press 3 if You do not know. | | | |
| R7. Are you currently working in a clinic or a health facility?  Press 1 for Yes  Press 2 for No | | | |
| R8. Are you currently in quarantine or admitted to a hospital or health facility?  Press 1 for Yes  Press 2 for No | | | |
| [PUBLIC HEALTH MESSAGE] COVID-19 is a new disease, caused by a virus.  The virus spreads mainly through the air when an infected person coughs, sneezes, or talks.  It can also be spread by touching or shaking hands with an infected person and then touching your mouth, nose, or eyes before washing your hands. Spread is more likely when people are in close contact with one another (within about 2 meters) or not wearing masks. | | | |
| [NO SYMPTOMS – SOCIAL DISTANCING]  To protect yourself and others from COVID-19, you should stay home as much as possible.  You should only leave your home for essential reasons such as obtaining food and water.  When outside your home, you should wear a face mask. You should also stay at least 2 meters away from other people, avoid groups and avoid public transit.  Wash your hands regularly with soap and water for 20 seconds (warm water is best if possible), especially after you have been outside your home. If this is not possible, use a hand sanitizer with 60% alcohol.  Avoid touching your face, particularly your mouth, eyes and nose, and especially when you are outside your home. | | | [If any of S1-S11 is Yes OR R6 is Yes]  [SYMPTOMS – SELF-ISOLATE]  Based on your answers, you should stay home and away from others. If possible, have a room that’s just for you. This will protect those around you.  You should avoid contact with others unless it is essential, such as when buying food or seeking medical care.  If you need to go out or be around others in the home, you should wear a face mask.  Wash your hands regularly with soap and warm water for 20 seconds, especially after you've been outside your home. If this is not possible, use a hand sanitizer with 60% alcohol.  Avoid touching your face, especially your mouth, eyes and nose.  Cover all coughs and sneezes and throw away used tissues or wash dirty handkerchiefs straight away. Do not sneeze or blow your nose into your hands.  Clean and disinfect surfaces in your home such as door handles each day.  Try to stay home and away from others for at least 14 days from when your symptoms first appeared.  Most people with COVID-19 symptoms will recover on their own without treatment from a doctor.  Eating well, drinking plenty of fluids, and getting a lot of rest can help you get better.  Call this toll free number to talk with a health provider from the Ministry of Health to discuss your symptoms and/or exposures and possible next steps: 0800-203-033. If you develop more symptoms or a serious symptom such as trouble breathing, persistent pain or pressure in your chest, confusion, or bluish lips or face, seek medical care immediately. |
| [STIGMA MITIGATION]  Anyone can contract COVID-19 including any age, race, tribe, nationality, occupation, as long as they are exposed to the virus.  If you are a contact of someone diagnosed with or suspected to have COVID-19, you should minimize contact with the community for at least 14 days or until you receive a negative COVID-19 test result.  After recovering from COVID-19, people can return to their family and community. Persons who have recovered from COVID-19 cannot transmit the infection to others. | | | |
| [FUTURE RESEARCH] Are you willing to be contacted by a research institution for additional research if you are eligible and selected? This may include COVID-19 testing, additional surveys, or an interview over the phone  Press 1 for Yes  Pres 2 for No. | | | |
| Thank you for taking the time to participate and listen! | | | |
| [If already completed the symptom checker this month]  Thank you for calling Dial-COVID. You are not eligible to participate at this time  as you participated this month already.  Please call again next month if you would like to take the survey and/or hear  information about COVID at that time. | | | |
